# Clinical manifestations of human leptospirosis: bacteria matter

**DOI:** 10.3389/fcimb.2023.1259599

**Published:** 2023-10-25

**Authors:** Jeanne Arline Rajaonarivelo, Anissa Desmoulin, Olivier Maillard, Louis Collet, Fiona Baudino, Marie-Christine Jaffar-Bandjee, Renaud Blondé, Loïc Raffray, Pablo Tortosa

**Affiliations:** ^1^ Unité Mixte de Recherche Processus Infectieux en Milieu Insulaire Tropical (UMR PIMIT), Université de La Réunion, CNRS 9192, INSERM 1187, IRD 249, La Réunion, France; ^2^ Service de Maladies Infectieuses, Centre Hospitalier Universitaire (CHU) de La Réunion Sites Sud, Réunion, France; ^3^ Service de Réanimation, Centre Hospitalier de Mayotte, Mayotte, France; ^4^ Department of Public Health and Research, Clinical Investigation Center (CIC), INSERM CIC 1410, CHU Réunion, Réunion, France; ^5^ Laboratoire de Microbiologie, Centre Hospitalier de Mayotte, Mayotte, France; ^6^ Laboratoire de Microbiologie, Centre Hospitalier Universitaire Felix Guyon, Réunion, France; ^7^ Service de Médecine Interne, Centre Hospitalier Universitaire Felix Guyon, Réunion, France

**Keywords:** host-pathogen interactions, virulence, Mayotte, human leptospirosis, one health, *Leptospira*

## Abstract

**Introduction:**

A high incidence of human leptospirosis is recorded on Mayotte, an oceanic island located in southwestern Indian Ocean, but the severity of the disease appears relatively mild in terms of mortality rate and admission to the intensive care unit. It has been proposed that mild leptospirosis may result from a limited virulence of some of the occurring *Leptospira* species to which the population is exposed.

**Methods:**

Clinical and biological data of patients admitted to the Centre Hospitalier de Mayotte were collected and the infecting *Leptospira* species were determined through molecular typing.

**Results:**

*Leptospira* interrogans was detected in the minority of admitted patients but most of these patients suffered from severe forms, with 50% admitted to intensive care unit and suffering from organ failures. Nineteen percent of patients infected with *Leptospira* borgpetersenii were admitted to the intensive care, with 13% displaying organ failures, and one patient died. *Leptospira* mayottensis was found in 28% of the patients and not a single severe case was observed.

**Discussion:**

The distribution of *Leptospira* species in patients was not different from that reported 10-15 years ago and bacterial genotypes were very closely related to those previously reported. These results highlight the importance of the diversity of pathogenic *Leptospira* circulating on Mayotte island and are in keeping with distinct outcome of the disease depending on the infecting *Leptospira*. Altogether, presented data support that the infecting *Leptospira* species is an important driver of disease severity in humans.

## Introduction

The integration of host-pathogen interactions in disease development is obviously essential to a comprehensive understanding of the epidemiology of a given disease. One way of highlighting drivers of virulence is to investigate host-pathogen interactions in which one of the two components is variable while the other remains relatively constant, such as an emerging infectious disease spreading rapidly throughout a wide geographical zone and hence affecting a diversity of ethnic groups. Alternatively, diseases relevant to tackle the importance of each compartment within host-pathogen interactions include those diseases caused by highly variable pathogens prevalent in a limited geographical location. We used this latter situation encountered on Mayotte, an oceanic island located in the western Indian Ocean, where the human population is heavily exposed to different species of pathogenic *Leptospira* (Bourhy et al., 2012; Tortosa et al., 2017).

Leptospirosis is a zoonotic disease caused by bacteria of the genus *Leptospira*, currently composed of more than 60 taxa including saprophytic and pathogenic species (Thibeaux et al., 2018; Vincent et al., 2019). Several wild mammals (e.g., rodents and bats) as well as pets and cattle can act as reservoirs, maintaining pathogenic *Leptospira* in their renal tubules and chronically shedding bacteria in their urine. Human infection mostly results from indirect contact with an environment contaminated with infected urine. The most common clinical signs of human leptospirosis include fever, headache, muscle aches and diarrhea, while some patients are asymptomatic. Severe infection, known as Weil’s disease, is related to multiple organs dysfunction (e.g., renal failure, severe hemorrhage, respiratory distress, neurological damage, etc.), which can lead to death in 1-10% of cases (Haake and Levett, 2015).

Leptospirosis has a worldwide distribution but highest incidence is recorded in tropical countries where warmer and wetter climatic conditions promote bacteria survival. On Mayotte island, the incidence rate is high (74.6 cases/100.000 population) while the mortality rate (0.6%) and admission to intensive care (4.6%) are relatively low (Subiros et al., 2017). On Mayotte, four species, namely *Leptospira borgpetersenii*, *Leptospira interrogans*, *Leptospira kirschneri* and *Leptospira mayottensis* are associated with human acute cases (Bourhy et al., 2012). Experimental infections using golden Syrian hamsters as models of acute infection have been conducted to measure the virulence of some of the regional pathogenic *Leptospira*, especially *L. mayottensis*, *L. borgpetersenii* and *L. interrogans*, isolated from tenrecs on Mayotte, bats from Madagascar and rats from Reunion island, respectively (Cordonin et al., 2019). The follow-up of weight gain and mortality rates in experimentally infected hamsters as well as tissue damages observed in kidneys and lungs, strongly suggest that the disease caused by *L. mayottensis* is distinctly less severe than that caused by *L. interrogans* (Cordonin et al., 2019). These data have led to a hypothesis according which the attenuated severity of the disease on Mayotte results from the bacterial species to which the local population is exposed. More specifically, an overall mild severity of the disease on Mayotte may result from a limited virulence of *L. mayottensis* and high severity of *L. interrogans* with a low proportion of human acute cases being infected by this latter pathogenic species.

To test this hypothesis, we recorded the clinical signs of patients diagnosed with leptospirosis at Mayotte Hospital over a two-year period and determined the infecting *Leptospira* species through molecular genotyping. Then, relations between the severity of clinical signs and the infecting *Leptospira* species were established in order to address whether the conclusions brought in by experimental infections are coherent with clinical and molecular data and support the importance of infecting *Leptospira* species on the severity of the disease.

## Materials and methods

### Patients inclusion

The study included a total of 221 patients hospitalized in the Centre Hospitalier de Mayotte from January 2018 to April 2020 and testing positive for leptospirosis through PCR on blood or urine, or ELISA serodiagnosis. All patients were informed on the use of the clinical data and blood samples for scientific research projects. The severity of clinical cases used the severe forms, presence of organ(s) failure(s), the admission to the Intensive Care Unit (ICU) and death. A severe form was defined by the presence of at least one of the following items: use of vasoactive drugs, and/or transfusion, and/or implementation of mechanical ventilation, and/or Renal Replacement Therapy (RRT). Targeted organs typical of leptospirosis included lung, kidney and liver. Lung failure was defined as need for oxygen or mechanical ventilation, kidney failure as a KDIGO score ≥ 2 (Kidney Disease Improving Global Outcomes evaluated with scale 0 to 3) or the need of RRT, and liver failure as total bilirubin ≥ 100 µM/L.

### *Leptospira* genotyping

*Leptospira* species was determined for patients whose blood samples were still available (59 out of 221). This was achieved directly from blood samples stored at the hospital in order to identify the *Leptospira* species through genotyping (see below) without any isolation step, notoriously challenging from frozen samples. A total of 200 µl nucleic acids (RNA/DNA) was extracted using a QIACUBE robot and Cador Pathogen kit (Qiagen, Valencia, California, USA) essentially using manufacturer’s instructions. Reverse transcription was performed on 10 µl of nucleic acids using the ProtoScript II Reverse Transcriptase and Random Primer 6 (New England BioLabs, Ipswich, Massachussets, USA) under the following thermal conditions: 70°C for 5 min (denaturation 1) then 17°C for 2 min, 25°C for 10 min (denaturation 2), 42°C for 50 min (annealing), and 65°C for 20 min (extension). Reverse transcription was carried out as it allows screening RNA (viral) and DNA pathogens from the same cDNA preparation, and because it increases the sensitivity of the *Leptospira* detection PCR scheme used herein, which targets the highly expressed 16S locus (Smythe et al., 2002). *Leptospira* were genotyped using Multi Locus Sequence Typing (MLST) scheme#3 (https://pubmlst.org/organisms/Leptospira-spp/) and the six following loci: *secY*, *rrs2*, *lipL32, adk*, *lipL41* and *icdA*, as this scheme has been widely used in different islands of Southwestern Indian ocean islands (Dietrich et al., 2018), including Mayotte (Lagadec et al., 2016), thus allowing comparing profiles with previously published sequence data. For this, 2 µl of cDNA were used as PCR template in a reaction mixture composed of 12.5 µl of GoTaq G2 Hot Start Green Master Mix (Promega, Madison, Wisconsin, USA), 0.5 µl (10 µM) of each primer (reverse and forward) and 9.5 µl of RNAse free water. PCR conditions were as follows: a first GoTaq activation step (95°C for 2 min) was followed by 45 cycles of denaturation (94°C for 30 sec), then annealing (58°C for 30 sec) and extension steps (72°C for 1 min). PCR thermal cycling was terminated by a final extension step (72°C for 5 min). Resulting amplicons were further Sanger sequenced (Genoscreen, Lille, France) on both strands. Chromatograms were manually aligned and edited using Geneious (9.1.8). Allelic numbers were determined using the Public databases for molecular typing and microbial genome diversity PubMLST (scheme#3).

### Statistical analysis

Categorical variables were described as numbers and percentages. Then, they were compared using Chi-square test or Fisher exact test, as appropriate. Statistical analyses were performed using SPSS software (IBM SPSS 23.0).

## Results

### Patients data

During the study period, a total of 221 patients tested positive for leptospirosis in the Centre Hospitalier de Mayotte. Mean age of the patients was 35.0 ( ± 12.1) years old and men represented 69% of the admitted patients.

### Genetic diversity of the infecting *Leptospira* strain

Out of the 221 positive patients, genotyping was attempted for 59 patients for which blood samples were available. DNA sequences of *Leptospira* species were obtained for 40 out of these 59 patients. Among the 40 patients with sequence data, *L. borgpetersenii* was most prevalent (43%, n = 17), followed by *L. mayotten*sis (28%, n *=* 11), *L. kirschneri* (20%, n = 8) and *L. interrogans* (10%, n = 4). Genotyping was carried out on all positive samples but did not allow full genotyping of all cases. Specifically, 22 sequences were obtained for *secY*, 35 for *rrs2*, 18 for *lipL32*, 13 for *adk*, 15 for *lipL41* and 7 for *icdA*. Among the 40 blood samples yielding PCR signals and sequences, two were sequenced on all six MLST loci and allowed identifying the infecting strain as *L. interrogans* ST138 serovar Pyrogenes. Even though we did not obtain full MLST for patients infected with *L. borgpetersenii*, *L. kirschneri* or *L. mayottensis*, we inferred the ST from the resulting partial allelic profiles. *Leptospira borgpetersenii* was represented by two allelic profiles, i.e., *secY*_48_, *rrs*_20_, *lipL32*_10_, *lipL41*_24_ corresponding to ST139 and *secY*_47_, *rrs_20_*, *lipL32*_10_, *adk*_65_ for which no ST was found on PubMLST database. Two allelic profiles were found for *L. kirschneri*, including *rrs*_12_, *lipL32*_11_, *lipL41*_21_, *icdA*_24_ corresponding to ST104 or ST158 while there was no ST correspondence for the second profile: *rrs*_12_, *lipL32*_12_, *lipL41*_16_, *icdA*_24_. As for *L. mayottensis*, *secY*_55_, *rrs*_27_, *adk*_74_ profile was found in 5 samples and was partially found in 6 additional samples (**Supplementary Table 1**).

### Clinical data associated with the different infecting *Leptospira* strains

We used clinical data collected from 40 patients from whom the infecting *Leptospira* species was identified. The severity of clinical cases varied according to the considered *Leptospira* species. In fact, the outcome of the disease appeared more severe for *L. interrogans* than for *L. borgpetersenii* infected patients. Among the severe cases infected with *L. interrogans* (n = 4), 50% were admitted in ICU (n = 2), with 50% (n = 2) requiring mechanical ventilation, 25% (n = 1) RRT, 50% (n = 2) the use of intravenous fluid therapy and vasoactive drugs (**Table 1**). Bleeding was recorded in half of the cases, with transfusion required in 25% (n = 1) of cases. Out of the 17 patients infected with *L. borgpetersenii*, 18% (n = 3) were admitted to intensive with 6% (n = 1) requiring mechanical ventilation, 12% (n = 2) RRT, 12% (n = 2) transfusion, and 6% (n = 1) vasoactive drugs. Noteworthily, one *L. borgpetersenii* infected patient deceased. None of the 8 patients infected with *L. kirschneri* was admitted to ICU but one patient required an intravenous fluid therapy and responded well to treatment. Not a single patient infected with *L. mayottensis* displayed severe manifestations of leptospirosis, representing 11 patients and 28% of the included patients with *Leptospira* species assignation. However, bleeding was reported for one patient without need for a transfusion and one patient required an intravenous fluid therapy. No deaths were observed for patients infected with *L. kirschneri* and *L. mayottensis*.

**Table 1:** Clinical severity according to the infecting *Leptospira* species.

	*Leptospira* species (N = 40)				
Clinical variables	*L. interrogans* n = 4 (10)	*L. borgpetersenii* n = 17 (43)	*L. kirschneri* n = 8 (20)	*L. mayottensis* n = 11 (28)	*P*
Severe forms, (%)	2/4 (50)	3/17 (18)	0	0	*
Admission to ICU, (%)	2/4 (50)	3/17 (18)	0	0	*
qSOFA ≥ 2, (%)	2/4 (50)	2/17 (12)	0	0	*
Intravenous fluid therapy, (%)	2/4 (50)	6/17 (35)	1/8 (12)	1/11 (9)	
Vasoactive drugs, (%)	2/4 (50)	1/17 (6)	0	0	
Bleeding, (%)	2/4 (50)	1/17 (6)	1/8 (12)	1/11 (9)	ns
Transfusion, (%)	1/4 (25)	2/17 (12)	0	0	
RRT, (%)	1/4 (25)	2/17 (12)	0	0	
Mechanical ventilation, (%)	2/4 (50)	1/17 (6)	0	0	
ARDS, (%)	0	0	0	0	
Organ failure, (%)	3/4 (75)	3/17 (19)	1/8 (12)	0	**
Liver, (%)	2/4 (50)	2/17 (13)	1/8 (11)	0	
Kidney, (%)	2/4 (50)	2/17 (13)	1/8 (11)	0	
Lung, (%)	2/4 (50)	2/17 (13)	0	0	
Death	0	1	0	0	

P-value = ** < 0.01; * < 0.05; ns, not significant.

ARDS: Acute Respiratory Distress Syndrome; ICU: Intensive Care Unit; qSOFA: quick Sequential (Sepsis-related Organ Failure Assessment); RRT: Renal Replacement Therapy.

Severe forms: use of vasoactive drug, and/or use of mechanical ventilation, and/or use of RRT, and/or use of transfusion.

KDIGO: Kidney Disease Improving Global Outcomes (scale 0 to 3).

Organ failure: Kidney: KDIGO score ≥ 2 or the need for Renal Replacement Therapy; Liver: total bilirubin ≥ 100 µM/L; Lung: need for oxygen therapy or mechanical ventilation.

## Discussion

This study aimed at investigating the importance of the infecting *Leptospira* species on the disease outcome in human acute cases. We focused on human leptospirosis on Mayotte since the island records high disease incidence and hosts a notable diversity of pathogenic *Leptospira* species. Partial or full genotyping of *Leptospira* confirms that the disease is caused by 4 distinct species: *L. borgpetersenii* (43%) was found in the majority of human cases followed by *L. mayottensis* (28%), *L. kirschneri* (20%) and *L. interrogans* (10%). A previous investigation from the Spirochetes national reference center (Bourhy et al., 2012) reported the same four *Leptospira* species within a panel of 95 patients sampled between 2007 and 2010. Although the distribution of each species was slightly modified when compared to this previous study reporting *L. borgpetersenii* (52.6%), *L. mayottensis* (15.8%) [formerly known as *L. borgpetersenii* group B (Bourhy et al., 2014)], *L. kirschneri* (23.2%) and *L. interrogans* (8.4%), the difference in species distribution reported herein is not significantly distinct from that reported over 10 years ago (Fisher exact test, p > 0.05).

When comparing allelic profiles reported in our study with those gathered from animals (Lagadec et al., 2016), *L. borgpetersenii* and *L. interrogans* sequences were identical or closely related with those previously detected in *Rattus rattus*, confirming this species as an important reservoir on the island. Similarly, alignments between *L. kirschneri* sequences from the present study and those identified as infecting humans by Lagadec et al., 2016, showed close identity, although the reservoir of this leptospiral species remains to be formally identified. Finally, *L. mayotten*sis genotype found herein corresponded to that reported in several *Tenrec ecaudatus* specimens sampled on Mayotte (Lagadec et al., 2016). Altogether, sequences data obtained in the frame of the present study were not different from those previously reported from patients (Bourhy et al., 2012) or domestic and wild animals (Bourhy et al., 2012; Lagadec et al., 2016). Hence, the epidemiological situation appears stable on the island over the last 10-15-year period. However, these data represent only 2 time points, and a comprehensive longitudinal survey is required to properly address the evolution of the epidemiology of leptospirosis on Mayotte.

We then compared the severity of symptoms in human acute cases according to distinct *Leptospira* genospecies infections. *Leptospira interrogans* caused a distinctly severe disease, with 2 out of the 4 included cases being admitted to intensive care and suffering from severe forms. *Leptospira interrogans* is a species of regional medical concern: this species is overwhelmingly dominant in human acute cases from Reunion island (Guernier et al., 2016), the other French administrated island in the Indian Ocean, and the only detected species in human acute cases on the island state of Seychelles (Biscornet et al., 2017), which records among the highest human incidence worldwide (Pappas et al., 2008). However, it is important to note that the *L. interrogans* STs prevalent in the different islands are diverse, with ST02, ST142 and ST143 found in Seychelles (Biscornet et al., 2017), ST02 and ST34 on Reunion island, while our typing reveals ST138 on Mayotte. Three patients out of 16 infected with *L. borgpetersenii* were admitted in the hospital’s intensive care and displayed organ failures. By contrast, no sign of severe cases was observed for any of the *L. kirschneri* and *L. mayottensis* infected patients, although representing half of all positive samples.

Of the 40 samples, only two complete MLST profiles were obtained particularly for *L. interrogans* allowing assignment to ST138, corresponding to serovar Pyrogenes. PCR failures through MLST using samples testing positive through RT-PCR is expected, as the RT-PCR used herein for *Leptospira* detection (Smythe et al., 2002) amplifies a much shorter DNA stretch (i.e. 55bp for *L. interrogans* serovar Lai Str. 56601) than do MLST PCRs (>400bp for all MLST *loci*). In addition, MLST has been developed using DNA from cosmopolitan *Leptospira* such as *L. interrogans*, which was the only species in our sample that could be fully genotyped. PCR failure, especially on *L. mayottensis*, which is endemic to Southwestern Indian Ocean region, may be thus related to potential mismatches in the primer regions, as previously suggested (Dietrich et al., 2018). While complete typing using blood samples instead of pure cultures is challenging, it can lead to full ST assignment for some of the qPCR positive samples as shown here for *L. interrogans*. However, *Leptospira* can still be identified to species using the *secY* gene, which has been shown to be highly discriminating at this level of taxonomic differentiation (Victoria et al., 2008). We could thus determine the infecting *Leptospira* species using *secY* marker (in addition to other markers when successfully sequenced), which allowed overlaying the symptoms of the included patients on the species of the infecting *Leptospira* and thus address the main research question.

Altogether, presented data support distinct clinical outcome depending on bacterial infection, with *L. interrogans* and *L. mayottensis* appearing associated with the most severe and mildest disease outcomes, respectively. This pattern is in keeping with experimental infections of golden Syrian hamsters used as an animal model of acute leptospirosis (Cordonin et al., 2019) as experimental infection with *L. interrogans* ST02 led to 80% mortality while *L. mayottensis* did not cause mortality nor have any impact on weight gain of experimentally infected hamsters. The infection with *L. interrogans* was also associated with severe lung and kidney tissue damages while tissue biopsies from *L. mayottensis* infected animals were mild or not different from the control animals. Hence, data presented herein further suggest that the apparent reduced severity of human leptospirosis on Mayotte results at least in part from the presence of *L. mayottensis*, leading to milder symptoms, and the rare occurrence of *L. interrogans* on the island, which leads to severe cases.

Although the severity of disease onset results from complex interactions involving host and microbial genomes as well as environmental determinants, presented results support an important role of leptospiral genome. Although reverse genetics is notoriously challenging on pathogenic *Leptospira* (Picardeau, 2018), a handful of studies have presented molecular features in the distinct *Leptospira* species, such as a reduced genome of *L. borgpetersenii* possibly correlating with a limited survival in the environment (Bulach et al., 2006) or genes unique to *L. interrogans* encoding protein families that are directly linked to the potential virulence of this species (Adler et al., 2011). These proteins are involved in endothelial and epithelial cell adhesion in the host and associated with the interaction of *Leptospira* with human plasma components, causing hemorrhages that are generally found in the severe forms of the disease (Mehrotra et al., 2017). Data presented herein bring further evidence that the severity of human leptospirosis is at least in part driven by the infecting *Leptospira*. The islands of Indian Ocean are an interesting environmental set-up as each island hosts different transmission chains, composed of different reservoir species and pathogenic *Leptospira* (Desvars et al., 2013; Guernier et al., 2016; Tortosa et al., 2017; Dietrich et al., 2018; Biscornet, 2020). Comprehensive investigations carried out under the One Health concept have identified some of the reservoirs of the occurring *Leptospira* species, with rats being the main reservoirs of *L. borgpetersenii* found in the majority of human acute cases, and tenrecs being uniquely shedding *L. mayottensis* (Lagadec et al., 2016). From a public health perspective, the distinct severity associated with the different *Leptospira* species, presented herein and indicated in a previous experimental infection experiment (Cordonin et al., 2019) suggest that control measures targeting one or other reservoir, such as rats or tenrecs, and possibly dogs that have been reported shedding *L. borgpetersenii* and *L. kirschneri*, will have different impact on human disease. Lastly, since most severe cases appear among *L. interrogans* infected patients, an early diagnosis allowing discriminating the leptospiral species such as High-Resolution Melting PCR (Naze et al., 2015) may help identifying patients with highest risk of developing severe forms on Mayotte.

## Data availability statement

The datasets presented in this study can be found in online repositories. The names of the repository/repositories and accession number(s) can be found below: https://www.ncbi.nlm.nih.gov/genbank/, OP776245-OP776260; https://www.ncbi.nlm.nih.gov/genbank/, OP782690-OP782699; https://www.ncbi.nlm.nih.gov/genbank/, OQ230549-OQ230575; https://www.ncbi.nlm.nih.gov/genbank/, OQ248114-OQ248138; https://www.ncbi.nlm.nih.gov/genbank/, OQ269484-OQ269510.

## Ethics statement

The studies involving humans were approved by Ethics committee of the Société de Pathologie Infectieuse de Langue Franç̧aise (SPILF), N°CER-MIT 2022-0505. The studies were conducted in accordance with the local legislation and institutional requirements. This retrospective study uses previous data and adheres to the French regulation MR-004 on clinical research (N°2206739). Within this framework, there is no need for formal notification, however a study information leaflet was provided to all patients.

## Author contributions

JR: Data curation, Investigation, Writing – original draft, Writing – review & editing. AD: Data curation, Formal analysis, Investigation, Writing – review & editing. OM: Data curation, Formal analysis, Writing – review & editing. LC: Resources, Writing – review & editing. FB: Investigation, Writing – review & editing. M-CJ-B: Resources, Writing – review & editing. RB: Resources, Writing – review & editing. LR: Methodology, Project administration, Resources, Supervision, Writing – review & editing. PT: Methodology, Project administration, Resources, Supervision, Writing – review & editing.
