# Supplementary material for: Clinical manifestations of human leptospirosis: bacteria matter
Source: Front Cell Infect Microbiol. 2023 Oct 25;13:1259599. doi: 10.3389/fcimb.2023.1259599 (PMC10635415; doi:10.3389/fcimb.2023.1259599)
Supplement: Supplementary file 1 [file Table_1.docx]

**Supplementary Table**

**TABLE 1 Allelic numbers for the six MLST loci, scheme #3. Missing allelic numbers correspond to negative PCRs and thus absence of sequence data.**

| **Patients labels** | ***Leptospira* species** | ***secY*** | ***rrs2*** | ***lipL32*** | ***adk*** | ***lipL41*** | ***icdA*** | **ST** |
| --- | --- | --- | --- | --- | --- | --- | --- | --- |
| AB0177 | [*L. borgpetersenii*](https://www.ncbi.nlm.nih.gov/Taxonomy/Browser/wwwtax.cgi?id=174) | 48 | 20 | 10 |  | 24 |  |  |
| AF0397 | [*L. borgpetersenii*](https://www.ncbi.nlm.nih.gov/Taxonomy/Browser/wwwtax.cgi?id=174) | 48 | 20 | 10 |  | 24 |  |  |
| CA1276 | [*L. borgpetersenii*](https://www.ncbi.nlm.nih.gov/Taxonomy/Browser/wwwtax.cgi?id=174) |  | 20 |  |  |  |  |  |
| DM1295 | [*L. borgpetersenii*](https://www.ncbi.nlm.nih.gov/Taxonomy/Browser/wwwtax.cgi?id=174) |  | 20 | 10 |  | 24 |  |  |
| EA0183 | [*L. borgpetersenii*](https://www.ncbi.nlm.nih.gov/Taxonomy/Browser/wwwtax.cgi?id=174) | 47 | 20 | 10 | 65 |  |  |  |
| FC0495 | [*L. borgpetersenii*](https://www.ncbi.nlm.nih.gov/Taxonomy/Browser/wwwtax.cgi?id=174) |  | 20 |  |  |  |  |  |
| FH1289 | [*L. borgpetersenii*](https://www.ncbi.nlm.nih.gov/Taxonomy/Browser/wwwtax.cgi?id=174) | 48 | 20 | 10 |  | 24 |  |  |
| HS0374 | [*L. borgpetersenii*](https://www.ncbi.nlm.nih.gov/Taxonomy/Browser/wwwtax.cgi?id=174) | 48 | 20 |  |  | 24 |  |  |
| IC1286 | [*L. borgpetersenii*](https://www.ncbi.nlm.nih.gov/Taxonomy/Browser/wwwtax.cgi?id=174) | 48 | 20 | 10 |  | 24 |  |  |
| IH1079 | [*L. borgpetersenii*](https://www.ncbi.nlm.nih.gov/Taxonomy/Browser/wwwtax.cgi?id=174) | 47 | 20 | 10 | 65 |  |  |  |
| IS0393 | [*L. borgpetersenii*](https://www.ncbi.nlm.nih.gov/Taxonomy/Browser/wwwtax.cgi?id=174) | 48 | 20 |  |  | 24 |  |  |
| MH0957 | [*L. borgpetersenii*](https://www.ncbi.nlm.nih.gov/Taxonomy/Browser/wwwtax.cgi?id=174) | 48 | 20 | 10 |  | 24 |  |  |
| MK1293 | [*L. borgpetersenii*](https://www.ncbi.nlm.nih.gov/Taxonomy/Browser/wwwtax.cgi?id=174) | 48 | 20 | 10 |  | 24 |  |  |
| SA0178 | [*L. borgpetersenii*](https://www.ncbi.nlm.nih.gov/Taxonomy/Browser/wwwtax.cgi?id=174) | 47 | 20 | 10 | 65 |  |  |  |
| YD1272 | [*L. borgpetersenii*](https://www.ncbi.nlm.nih.gov/Taxonomy/Browser/wwwtax.cgi?id=174) |  | 20 |  |  |  |  |  |
| YH0179 | [*L. borgpetersenii*](https://www.ncbi.nlm.nih.gov/Taxonomy/Browser/wwwtax.cgi?id=174) |  | 20 | 10 | 65 |  |  |  |
| ZS0484 | [*L. borgpetersenii*](https://www.ncbi.nlm.nih.gov/Taxonomy/Browser/wwwtax.cgi?id=174) |  | 20 |  |  |  |  |  |
| AM0787 | *L. interrogans* | 58 |  |  |  |  |  |  |
| BA1272 | *L. interrogans* | 58 | 2 | 2 | 5 | 2 | 22 | 138 |
| IL0982 | *L. interrogans* | 58 | 2 | 2 | 5 | 2 | 22 | 138 |
| YA1202 | *L. interrogans* |  | 2 | 2 | 5 |  | 22 |  |
| AM081987 | [*L. kirschneri*](https://www.ncbi.nlm.nih.gov/Taxonomy/Browser/wwwtax.cgi?id=29507) |  | 12 |  |  |  |  |  |
| AN1001 | *L. kirschneri* |  | 12 |  |  |  |  |  |
| IM171 | [*L. kirschneri*](https://www.ncbi.nlm.nih.gov/Taxonomy/Browser/wwwtax.cgi?id=29507) |  | 12 | 12 |  | 16 | 24 |  |
| MF0700 | [*L. kirschneri*](https://www.ncbi.nlm.nih.gov/Taxonomy/Browser/wwwtax.cgi?id=29507) |  | 12 |  |  | 16 | 24 |  |
| ND1278 | [*L. kirschneri*](https://www.ncbi.nlm.nih.gov/Taxonomy/Browser/wwwtax.cgi?id=29507) |  |  | 11 |  |  |  |  |
| NM0177 | [*L. kirschneri*](https://www.ncbi.nlm.nih.gov/Taxonomy/Browser/wwwtax.cgi?id=29507) |  | 12 |  |  | 21 | 24 |  |
| NO1267 | [*L. kirschneri*](https://www.ncbi.nlm.nih.gov/Taxonomy/Browser/wwwtax.cgi?id=29507) |  |  | 11 |  |  |  |  |
| SA0184 | [*L. kirschneri*](https://www.ncbi.nlm.nih.gov/Taxonomy/Browser/wwwtax.cgi?id=29507) |  | 12 | 11 |  | 21 | 24 |  |
| AL0876 | [*L. mayottensis*](https://www.ncbi.nlm.nih.gov/Taxonomy/Browser/wwwtax.cgi?id=1137606) |  | 27 |  |  |  |  |  |
| AZ0169 | [*L. mayottensis*](https://www.ncbi.nlm.nih.gov/Taxonomy/Browser/wwwtax.cgi?id=1137606) | 55 | 27 |  | 74 |  |  |  |
| BS0691 | [*L. mayottensis*](https://www.ncbi.nlm.nih.gov/Taxonomy/Browser/wwwtax.cgi?id=1137606) |  | 27 |  | 74 |  |  |  |
| MD1186 | *L. mayottensis* | 55 |  |  |  |  |  |  |
| MF1295 | [*L. mayottensis*](https://www.ncbi.nlm.nih.gov/Taxonomy/Browser/wwwtax.cgi?id=1137606) | 55 | 27 |  | 74 |  |  |  |
| MM1290 | [*L. mayottensis*](https://www.ncbi.nlm.nih.gov/Taxonomy/Browser/wwwtax.cgi?id=1137606) |  | 27 |  |  |  |  |  |
| MY0894 | [*L. mayottensis*](https://www.ncbi.nlm.nih.gov/Taxonomy/Browser/wwwtax.cgi?id=1137606) | 55 |  |  |  |  |  |  |
| NN0576 | [*L. mayottensis*](https://www.ncbi.nlm.nih.gov/Taxonomy/Browser/wwwtax.cgi?id=1137606) | 55 | 27 |  | 74 |  |  |  |
| SH0783 | [*L. mayottensis*](https://www.ncbi.nlm.nih.gov/Taxonomy/Browser/wwwtax.cgi?id=1137606) | 55 | 27 |  | 74 |  |  |  |
| SH1272 | *L. mayottensis* | 55 | 27 |  |  |  |  |  |
| SO0197 | *L. mayottensis* | 55 | 27 |  | 74 |  |  |  |
